# DNA Barcoding of *Bemisia tabaci* Complex (Hemiptera: Aleyrodidae) Reveals Southerly Expansion of the Dominant Whitefly Species on Cotton in Pakistan

**DOI:** 10.1371/journal.pone.0104485

**Published:** 2014-08-06

**Authors:** Muhammad Ashfaq, Paul D. N. Hebert, M. Sajjad Mirza, Arif M. Khan, Shahid Mansoor, Ghulam S. Shah, Yusuf Zafar

**Affiliations:** 1 Biodiversity Institute of Ontario, University of Guelph, Guelph, Ontario, Canada; 2 National Institute for Biotechnology and Genetic Engineering, Faisalabad, Pakistan; 3 Entomology Section, Agricultural Research Institute, Tandojam, Pakistan; 4 Agri & Biotech Division, Pakistan Atomic Energy Commission, Islamabad, Pakistan; Zhejiang University, China

## Abstract

**Background:**

Although whiteflies (*Bemisia tabaci* complex) are an important pest of cotton in Pakistan, its taxonomic diversity is poorly understood. As DNA barcoding is an effective tool for resolving species complexes and analyzing species distributions, we used this approach to analyze genetic diversity in the *B. tabaci* complex and map the distribution of *B. tabaci* lineages in cotton growing areas of Pakistan.

**Methods/Principal Findings:**

Sequence diversity in the DNA barcode region (mtCOI-5′) was examined in 593 whiteflies from Pakistan to determine the number of whitefly species and their distributions in the cotton-growing areas of Punjab and Sindh provinces. These new records were integrated with another 173 barcode sequences for *B. tabaci*, most from India, to better understand regional whitefly diversity. The Barcode Index Number (BIN) System assigned the 766 sequences to 15 BINs, including nine from Pakistan. Representative specimens of each Pakistan BIN were analyzed for mtCOI-3′ to allow their assignment to one of the putative species in the *B. tabaci* complex recognized on the basis of sequence variation in this gene region. This analysis revealed the presence of Asia II 1, Middle East-Asia Minor 1, Asia 1, Asia II 5, Asia II 7, and a new lineage “Pakistan”. The first two taxa were found in both Punjab and Sindh, but Asia 1 was only detected in Sindh, while Asia II 5, Asia II 7 and “Pakistan” were only present in Punjab. The haplotype networks showed that most haplotypes of Asia II 1, a species implicated in transmission of the cotton leaf curl virus, occurred in both India and Pakistan.

**Conclusions:**

DNA barcodes successfully discriminated cryptic species in *B. tabaci* complex. The dominant haplotypes in the *B. tabaci* complex were shared by India and Pakistan. Asia II 1 was previously restricted to Punjab, but is now the dominant lineage in southern Sindh; its southward spread may have serious implications for cotton plantations in this region.

## Introduction

The whitefly, *Bemisia tabaci* (Gennadius) (Hemiptera: Aleyrodidae) is now recognized as a cryptic species complex [1], [2] composed of at least 34 [3]–[7] morphologically indistinguishable, but reproductively isolated species [8], [9]. Members of the complex damage a wide range of agricultural and horticultural crops through both their feeding activity and their role in the transmission of plant viruses. Three members of the complex, Asia 1, Asia II 1, and Middle East-Asia Minor 1 (MEAM 1), have been previously identified from Pakistan where they are associated with the transmission of cotton leaf curl disease (CLCuD) which causes a significant reduction in yield [10], [11]. The severity of CLCuD varies across Pakistan with higher losses in central (Punjab) than southern (Sindh) Pakistan [12]. There has been a continuing debate as to the identity of the whitefly lineages in these regions and whether differences in the vector pool account for the differing levels of infection on cotton plants from these provinces. Ahmed et al. [11] found that MEAM 1 was restricted to Sindh and Asia II 1 to the Punjab, whereas Asia 1 was found in both regions. Because Asia II 1 was associated with a higher incidence of CLCuD in both Punjab and northeastern India [11], [13], it is thought to play an important role in the transmission of this disease.

A number of DNA-based techniques have been used to identify species of whiteflies [14]–[19]. However, most of our understanding of genetic relationships in the *B. tabaci* complex comes from the examination of sequence diversity in the mitochondrial cytochrome *c* oxidase I (COI) gene. Frohlich et al. [20] were the first to use COI to distinguish lineages of *B. tabaci*, employing the 3′ end of the gene, a standard adopted by subsequent investigators with the result that 383 different haplotypes have now been identified for this gene region [21]. Analysis of these haplotypes has revealed the presence of 28 distinct networks plus seven unconnected haplotypes [22]. Of these networks, 24 correspond to the putative species identified by Dinsdale et al. [3]. Researchers have shown that different species in the *B. tabaci* complex have varied global invasion histories [22] and that these lineages have differential roles in transmitting leaf curl disease to various crops [23], [24].

Prior studies have shown that local differences in the abundance of different species within the *B. tabaci* complex are due, at least in part, to competition [25]–[28] with one member of the complex often displacing another [26], [29], [30]. Furthermore, shifts in both distribution and abundance can occur rapidly [26], [29] as the invading species gains an advantage over the established species by asymmetric mating interactions [31]. Such displacements or expansion in species ranges have important implications for pest and pest-vectored disease management strategies [32]. Concerns [33] have already been raised in relation to the spread of varied members of the *B. tabaci* complex and the viruses they transmit.

The rise of DNA barcoding as a tool for species identification across the animal kingdom [34]–[36] has led to a database that now includes 2.9 million COI-5′ sequence (barcode) records derived from more than 318K animal species. Efforts are underway to construct comprehensive DNA barcode reference libraries for various animal groups including pest species [37]–[40]. These libraries not only aid the documentation of biodiversity [41], but also facilitate the identification of invasive species [42], [43]. However, because little sequence analysis has been directed toward the barcode region in *B. tabaci*, there is no ‘translation table’ to connect the lineages of this species which have been recognized based on their COI-3′ sequence with their COI-5′ counterparts [43].

In this study, we use DNA barcodes to discriminate the lineages of *B. tabaci* found in India and Pakistan, reveal their genetic diversity and subsequently test if their distributions have shifted in the cotton-growing areas of Punjab and Sindh since a study in 2007–2009 [11]. We also employ barcodes to separate species of the *B. tabaci* complex and begin construction of the ‘translation’ matrix from COI-3′ to COI-5′. Because barcode reference libraries enable species identification, the study provides insights into the diversity, movement, and distributional patterns of species in the *B. tabaci* complex in the region.

## Materials and Methods

### Ethics Statement

No specific permissions were required for this study. The study did not involve endangered or protected species.

### Collection of whiteflies

Adult whiteflies were collected by sampling 255 sites within Punjab and Sindh from 2010 to 2013. Sampling followed protocols outlined by Ahmed et al. [11]. GPS coordinates were recorded [Table S1] and collection localities and species distributions were mapped using an online tool (www.simplemappr.net). Samples were collected using an aspirator, then transferred to 95% ethanol and stored at −20°C until analysis. Two to three whiteflies were chosen from each collection site, producing a total of 649 specimens for barcode analysis. Individual whiteflies were labeled, assigned specimen numbers and photographed. Specimen data along with the collection information were added to the project MAWFL (Whitefly Species Complex of Pakistan) in BOLD (www.boldsystems.org), the Barcode of Life Data System [44]. All barcode compliant sequences from *B. tabaci* available in GenBank (173) were also analyzed to gain a better understanding of the global patterns of barcode diversity in *B. tabaci*.

### DNA isolation

Genomic DNA was extracted from most specimens at the Canadian Centre for DNA Barcoding using the protocol described by Porco et al. [45], but a few specimens processed early in the study were analyzed using methods outlined by Erlandson et al. [46]. In brief, these specimens were homogenized individually in 250 µl of Lifton buffer, proteins were precipitated by potassium acetate, and DNA was then purified by phenol-chloroform extraction. Precipitated DNA pellets were resuspended in 50 µL of sterile ddH_2_O with 0.5 µL of 10 mg RNase A/mL.

### mtCOI PCR amplification and sequencing

Amplification of the barcode region (COI-5′) was performed with primer pair LepF2_t1 (TGTAAAACGACGGCCAGTAATCATAARGATATYGG)/LepR1 (TAAACTTCTGGATGTCCAAAAAATCA) following the PCR conditions; 94°C (1 min), 5 cycles of 94°C (40 s), 45°C (40 s), 72°C (1 min); 35 cycles of 94°C (40 s), 51°C (40 s), 72°C (1 min) and final extension of 72°C (5 min). Amplification of COI-3′ was performed with primer pair C1-J-2183 (CAACATTTATTTTGATTTTTTGG)/TL2-N-3014 (TCCAATGCACTAATCTGCCATATTA) [47] following the PCR conditions; 94°C (1 min), 40 cycles of 94°C (40 s), 48°C (40 s), 72°C (1 min) and final extension of 72°C (5 min). PCRs were carried out in 12.5 µL reactions containing standard PCR ingredients and 2 µL of DNA template. PCR products were analyzed on 2% agarose E-gel 96 system (Invitrogen Inc.). Amplicons were sequenced bidirectionally using the BigDye Terminator Cycle Sequencing Kit (v3.1) (Applied Biosystems) on an Applied Biosystems 3730XL DNA Analyzer. The forward and reverse sequences were assembled, aligned and edited using CodonCode Aligner (CodonCode Corporation, USA) and submitted to BOLD. Sequences were also inspected and translated in MEGA V5 [48] to verify that they were free of stop codons and gaps. All sequences generated in this study and their GenBank accession numbers (Table S1) are accessible on BOLD in the dataset DS-MAWFL.

### Cryptic species discrimination using Barcode Index Numbers (BINs)

Past researchers have often assigned specimens to operational taxonomic units (OTUs) in cases where morphological identifications are difficult [49], [50]. Although this approach has sometimes been criticized [51], its general value has been accepted [52], [53]. Ratnasingham and Hebert [54] recently developed the Barcode Index Number (BIN) system which adds important new functionalities. Since its inception, the BIN system has been used as a species-level taxonomic registry for various animal groups [55]–[57] and has aided the discovery of new species [58]. As a result, all *B. tabaci* sequences in this study were assigned to a BIN.

### Analysis of barcode data from BOLD/GenBank

All barcode data for *B. tabaci* available on BOLD and GenBank were assembled to assess the growth in coverage since the most recent report [43]. There are now 766 barcode records for *B. tabaci* on BOLD (inclusive of this study and 173 accessions on GenBank, all of which were imported to BOLD (accessed December 17, 2013)). These barcode records were used in a combined analysis with the Pakistan data to determine the number of COI-5′ lineages in the *B. tabaci* complex and to ascertain genetic distances among these lineages.

### Cryptic species identification using mtCOI-3′

This study does not evaluate evolutionary relations in the *B. tabaci* complex as this topic has seen extensive work [3], [21], [59], although reassessment of the number of its constituent species continues [7]. Instead, we construct a barcode reference library and determine the number and distributional patterns of whitefly lineages in Pakistan. Dinsdale et al. [3] used a 3.5% (K2P) sequence threshold for COI-3′ to delimit different members of the *B. tabaci* complex. Boykin et al. [60] subsequently compared the results from this approach with those obtained with four other delimitation methods (Rosenberg’s reciprocal monophyly, Rodrigo’s (P(randomly distinct)), the genealogical sorting index, and general mixed Yule-coalescent) and found that all recognized the same number of genetic lineages. Since the existing nomenclature for members of the *B. tabaci* complex is based on sequence diversity in COI-3′, we also sequenced this gene region for representative specimens from each COI-5′ BIN detected in our study. This enabled their assignment to one of the species recognized on the basis of COI-3′ sequence variation by comparing each COI-3′ sequence to the reference sequences for the species in the *B. tabaci* complex [3], [6], [61]. Reference COI-3′ sequences were obtained from the global *Bemisia* dataset [6].

### Distance and phylogenetic analysis

ClustalW nucleotide sequence alignments [62] and pairwise (K2P) distance analysis were performed using MEGA5. The online version of ABGD [63] was used to generate distance histograms and distance ranks. Because the BINs [54] and the putative species [3] of *B. tabaci* were represented by variable number of sequences, a consensus sequence for each BIN or species was obtained using the ‘Consensus Barcode Generator’ function of TaxonDNA [64]. Consensus sequences were used in Bayesian inference (BI) and BI trees were obtained using MrBayes v3.2.0 [65] and the Markov Chain Monte Carlo (MCMC) technique. The data was partitioned in two ways; i) a single partition with parameters estimated across all codon positions, ii) a codon-partition in which each codon position was allowed different parameter estimates. The analyses were run for 10 million generations with sampling every 1,000 generations. We modeled the evolution of sequences according to the GTR+Γ model independently for the two partitions using the ‘‘unlink’’ command in MrBayes. The model selection was made using FindModel (www.hiv.lanl.gov/cgi-bin/findmodel/findmodel.cgi). Bayesian posterior probabilities were calculated from the sample points once the MCMC algorithm began to converge. Convergence was determined when the standard deviation of split frequencies went below 0.02 and the PSRF (potential scale reduction factor) approached 1, and both runs had properly converged to a stationary distribution after the burn-in stage (discarded the first 25% of samples). The trees generated through this process were visualized using FigTree v1.4.0. Two whitefly species, *Trialeurodes vaporariorum* (AY521265) and *Bemisia afer* (EU825777) were included in the analysis as outgroups. The parsimony analysis was performed using the same datasets with the TNT (Tree analyses using New Technologies) v1.1 ( Willi Hennig Society Edition) [66]. The analysis utilized New Technology heuristic searches [67] implemented in the program which consisted of Tree Fusion, Ratchet, Tree Drifting and Sectorial searches performed, with default parameters applied, until the most parsimonious tree was found 1000 times. Gaps in the molecular data were treated as missing characters. All characters were treated as unordered and equally weighted and the robustness of the reconstructed phylogenies was evaluated by bootstrap analysis (500 replicates).

### Genetic diversity, haplotype and distribution analysis

Genetic diversity indices and neutrality tests (Fu’s *Fs* [68] and Tajima’s *D* [69]) were performed in DnaSP v5.10.01 [70]. ClustalW aligned sequences from MEGA5 were exported as MEGA files and barcode haplotypes for each *B. tabaci* species from Pakistan (Asia II 1, Asia II5, Asia II 7, Asia 1, MEAM 1) were calculated using Arlequin v.3.5 [71]. For each species, a minimum spanning tree (MST) based on the number of nucleotide differences between haplotypes was constructed using a distance matrix from Arlequin in Hapstar v. 0.6 [72] to visualize the network of interrelationships between the haplotypes. Distributions of the identified species in the *B. tabaci* complex were mapped using GPS coordinates and an online tool (www.simplemappr.net).

## Results

### DNA barcode analysis of *B. tabaci*

**Figure 1:**
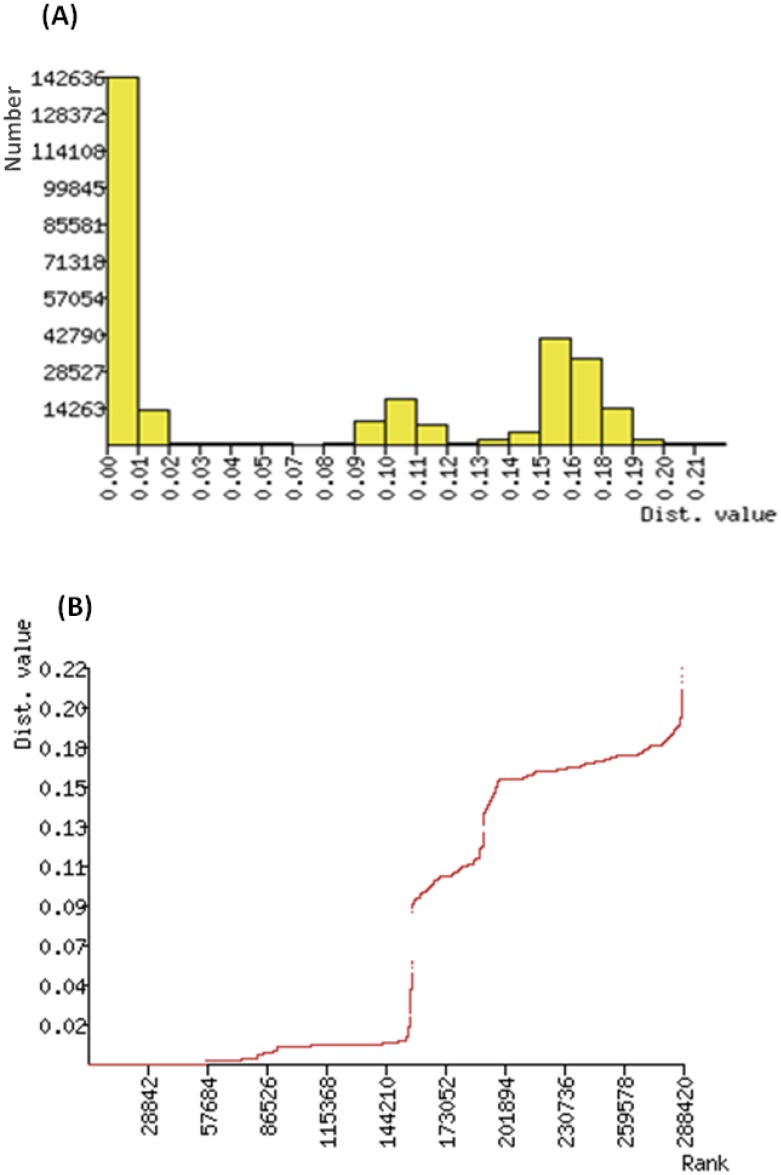
Histogram (A) and ranked (B) pairwise (K2P) distances among 762 barcode sequences of *B. tabaci* complex.

Barcode compliant sequences (>500 bp of COI-5′) were recovered from 589 of the 649 specimens (90%) from Pakistan (an additional four sequences recovered were <500 bp). Another 173 sequences for *B. tabaci* were added to the analysis including 146 from India, and 27 from Australia, Canada and Japan. Pairwise distances (K2P) among the sequences from Pakistan ranged from 0.0%–19.9% with a mean of 4%. The BIN system assigned these sequences to nine BINs. The pairwise distances in the combined (Pakistan + GenBank) *B. tabaci* sequences ranged from 0%–20% (mean = 8%). Maximum intraspecific (within BIN) distances ranged from 0.0%–2.3%, while the nearest-neighbor (NN) distances ranged from 1% to 16.7% (Table 1). Except for an intraspecific distance of 2.3% in AAM1247, the maximum intraspecific distances were less than 2% for each BIN (Table 1). The combined *B. tabaci* barcodes were assigned to 15 unique BINs (Table 1). Ten BINs derived from India, and seven of these were also detected from Pakistan (Table 1). Two other BINs (ACF7855, ABX2616) were only detected in Pakistan, while three (AAM1243, AAM1248, ACD5051) were exclusively from India. Two BINs (AAG4846, ACD4212) have not previously been reported from the Indo-Pakistan region (Table 1), while the origin of one BIN (AAA4495) is unknown. Histograms of sequence divergence values and ranked distances among barcode sequences in *B. tabaci* complex are shown in Fig. 1. There was a clear gap between the intraspecific and interspecific K2P distances with a majority of the intraspecific distances falling well below 2%.

**Table 1:** COI-5′ (BIN)/COI-3′ (species) translation, BIN distances and host plants of the members of the *Bemisia tabaci* complex.

Analysis group		*n* (>500 bp)	Max dist (K2P)	Dist to NN BIN	Host plants	Country of origin
BIN [54]	Dinsdale species [3]					
–	*B. tabaci* complex	762	19.7	-		
AAM1243	–	14	0.8	9.3	okra, common bean,cowpea, cotton, sunflower,tomato, sweet potato,brinjal	India
AAM1244	Asia 1	77	1.2	13.5	brinjal, cotton, cowpea,tomato, sunflower	India, Pakistan
AAM1245	Asia II 5	22	1.4	8.1	tomato, mulberry, cassava,groundnut, wild colocasia,cucurbita, blackgram,tobacco, cotton, Indiannettle, ipomea	India, Pakistan
AAM1246	–	2	0.8	2.9	Cotton	India, Pakistan
AAM1247	Asia II 1	551	2.3	2.9	cotton, brinjal, blackgram,tomato, mulberry, okra,cucurbit, pumpkin,zucchini, bottle gourd,chillies, sesame, clusterbean, unidentified weed	India, Pakistan
AAM1248	–	3	0.6	1.6	tobacco, sunflower, spiderflower	India
AAT8875	MEAM1	47	1.0	8.8	cotton, cabbage,cauliflower, unidentifiedweed	Australia, Canada, India, Pakistan
AAA4495	–	2	0.0	14.4	unknown	GenBank, N/A
AAG4846		20	1.9	8.8	unknown	Canada, Australia, Japan
ACD4214	–	2	0.0	16.7	unknown	Japan
ACD5051	–	3	0.0	9.4	cotton	India
ACE6289	Asia II 7	6	0.4	1.3	brinjal, Malaise collection	India, Pakistan
ACF2778	Asia II 7	3	0.5	1.0	cotton, white tamarind	India, Pakistan
ACF7855	Asia II 7	8	0.3	1.0	Malaise collection	Pakistan
ABX2616	“Pakistan”	1	–	14.3	Malaise collection	Pakistan

NN = nearest neighbour; BIN = Barcode Index Number.

### Barcode and COI-3′ connection of *B. tabaci*

**Figure 2:**
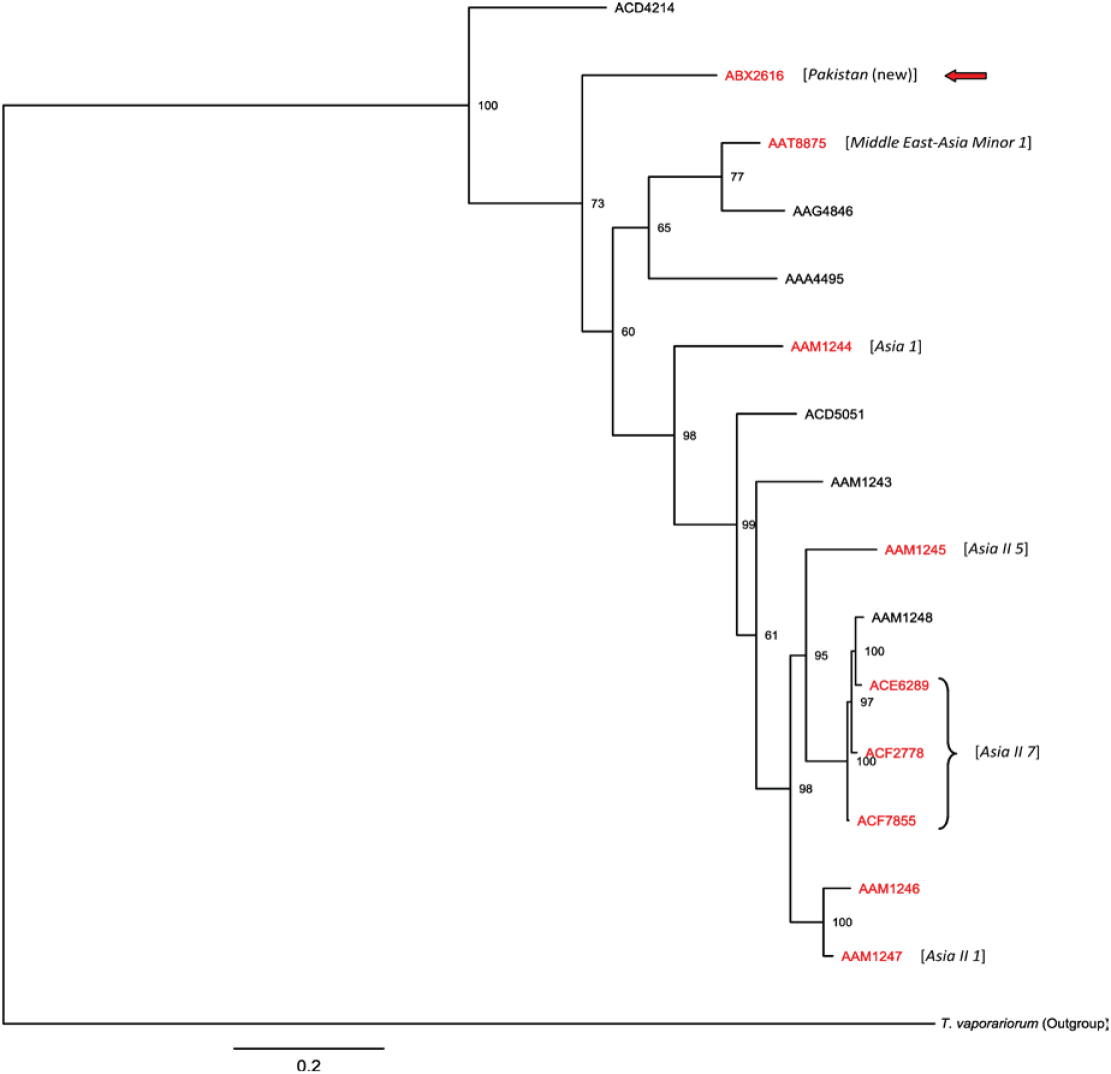
BIN-based phylogenetic analysis of *B. tabaci* complex. The tree was estimated using Bayesian inference. Posterior probabilities are indicated at nodes. Dinsdale species [3] identified from Pakistan are shown (in square brackets) next to their associated BINs (in red).

Analysis of the COI-3′ sequences from seven of the *B. tabaci* BINs from Pakistan showed their correspondence with five of the 34 putative species [6] of *B. tabaci*: Asia 1, Asia II 1, Asia II 5, Asia II 7 and MEAM 1 (Table 1, Fig. 2). Maximum distance among COI-3′ sequences of three BINs (ACE6289, ACF2778, ACF78) was less than 1.3%, and following the distance limit (3.5%) for *B. tabaci* species differentiation set by Dinsdale et al. [3], these BINs were assigned to the same species, Asia II 7 (Table 1, Fig. 2). The COI-3′ sequence of the BIN: ABX2616 is extremely divergent from any known clade, showing 13.7% divergence from the nearest neighbour (Table S2) in the existing whitefly databases [3], [6]. Because this genotype substantially exceeds the 3.5% sequence threshold employed for taxon recognition, this lineage represents a new addition to the *B. tabaci* complex which is named as “Pakistan”. The K2P distances among COI-3′ consensus sequences of 34 putative species in the *Bemisia* database [6] ranged between 1.3–22.7% and the new putative species “Pakistan” showed a NN distance of 13.7% (Table S2). Barcode sequences for the other eight BINs, including AAM1246 from Pakistan were obtained from GenBank and their corresponding COI-3′ sequences were unavailable, preventing their connection with Dinsdale nomenclature [3].

**Figure 3:**
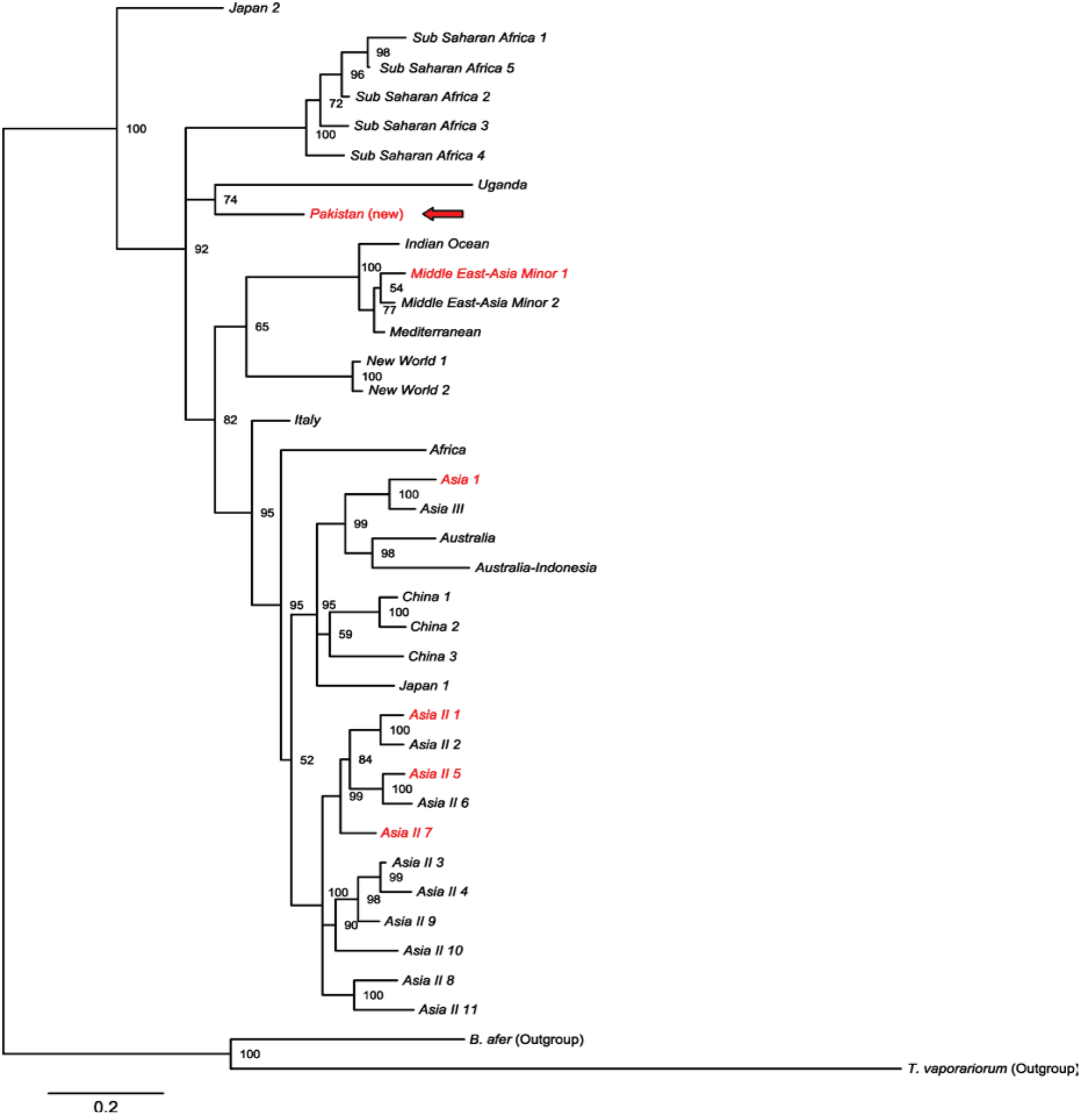
Phylogenetic relationship of the new *B. tabaci* lineage “Pakistan” (indicated by an arrow) with those reported by Dinsdale et al. [3] and De Barro and Boykin [6]. The tree was estimated using Bayesian inference. Posterior probabilities are shown next to the branches. Species also detected in Pakistan are in red.

**Figure 4:**
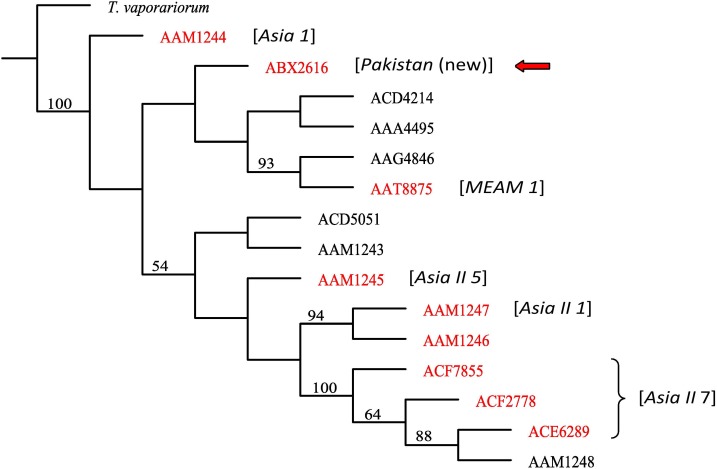
Single MPT inferred from the barcode sequences from *B. tabaci* Complex. Bootstrap values are shown above the branches (values <50% not shown). Dinsdale species [3] identified from Pakistan are shown (in square brackets) next to their associated BINs (in red).

**Figure 5:**
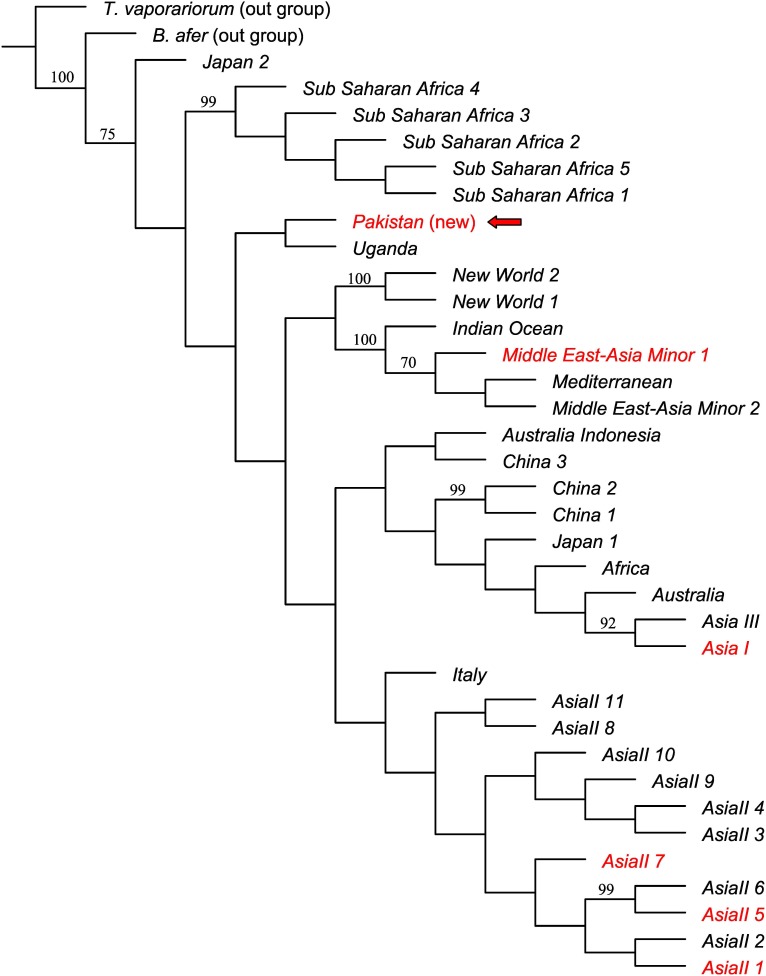
Single MPT showing position of the new *B. tabaci* lineage “Pakistan” (indicated by an arrow). Analysis included all the *B. tabaci* COI-3′ sequences from the global *Bemisia* dataset [6] and from Dinsdale et al. [3]. Bootstrap values are shown above the branches (values <50% not shown). Species also detected in Pakistan are in red.

The phylogenetic tree of *B. tabaci* BINs showed a close relationship among three species of Asia II group (Asia II 1, Asia II 5, Asia II 7) (Fig. 2) which clustered together with a 98% posterior probability (PP). Barcodes of Asia II 7 were assigned to three BINs (Fig. 2, Table 1) indicating the presence of considerable sequence variation in this taxon. Both the barcode and COI-3′ sequences of the BIN: ABX2616 did not match any sequence in the available databases (14.3% divergence from NN barcode and 13.7% from NN COI-3′ (Table S2)) and thus it was proposed as a new lineage “Pakistan”. Barcode-based BI showed that this lineage was phylogenetically closer to MEAM 1 than to the species in the Asia group (Asia I, Asia II) (Fig. 2). DNA barcode analysis of the *B. tabaci* complex from sites around the world is not complete, so we used CO1-3′ sequences to determine the position and phylogenetic relationship of the new *B. tabaci* lineage “Pakistan” (ABX2616). Taken as a whole, the COI-3′ based phylogenetic tree of *B. tabaci* (Fig. 3) was similar in topology to those generated by other researchers [3], [6], [7]. The “Pakistan” lineage was sister to the putative species “Uganda” (PP = 0.75) and formed a separate clade between the New World and the Subsaharan Africa species (Fig. 3). The relationship of the new lineage “Pakistan” with other members of the *B. tabaci* complex was further ascertained by the parsimony analysis. The most parsimonious trees (MPT) for the barcode (Fig. 4) and the COI-3′ (Fig. 5) sequences showed that the topology retrieved under parsimony analysis was not in conflict with that obtained using Bayesian Inference. Thus both methods of phylogenetic reconstruction placed the putative species “Pakistan” as a distinct clade sister to “Uganda”.

### Genetic diversity and species distributions

**Figure 6:**
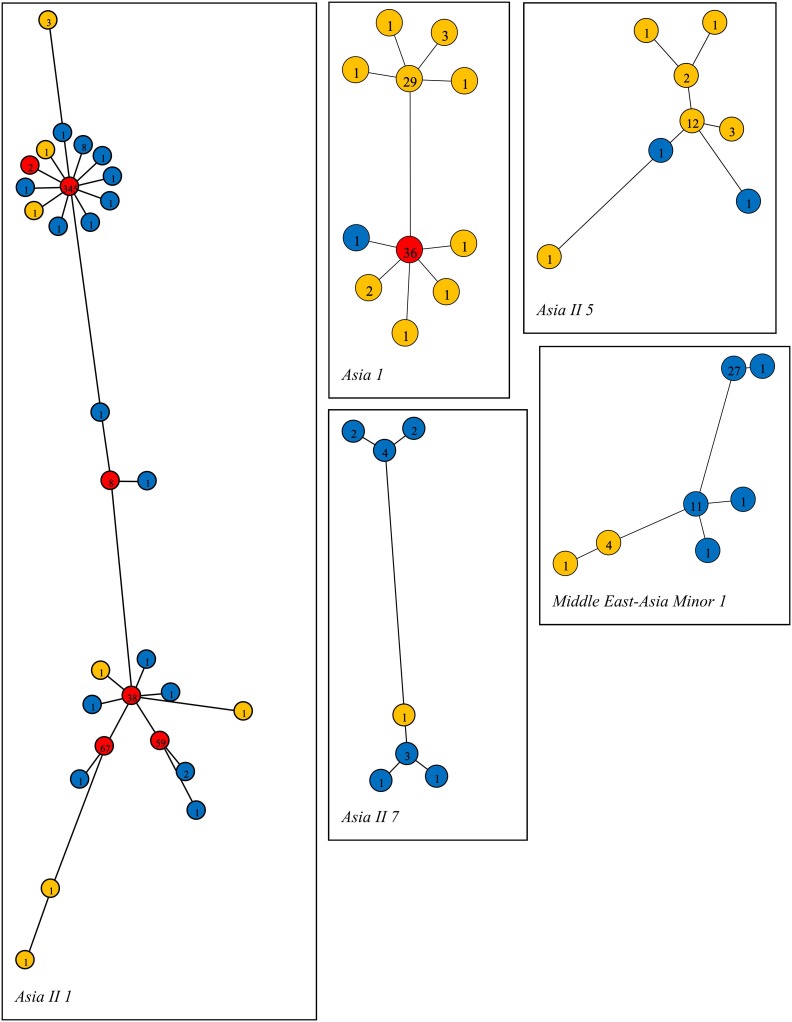
Barcode haplotype networks of *B. tabaci* species identified from Pakistan by corresponding COI-3′ sequences and named following Dinsdale nomenclature [3]. Barcode sequences of *B. tabaci* species shared between India and Pakistan were also included. Numbers in circles show the haplotype frequencies. Blue and yellow circles indicate the detection of a haplotype solely in Pakistan or India, respectively, while red circles indicate haplotypes present in both countries.

The genetic diversity indices are presented in Table 2. The average number of pairwise nucleotide differences, k, and nucleotide diversity, π, were relatively higher in Asia 1 (n = 77) and Asia II 7 (n = 14) than in Asia II 1 (n = 551), the most common species in the region. Haplotype network analysis revealed 29 haplotypes among the 551 sequences of Asia II 1 from Pakistan and India (Fig. 6). One haplotype was dominant (63%), occuring in all populations from both countries and in all cotton-growing areas of Pakistan. Three other haplotypes with a relatively high frequency (>7%) and two with a low frequency (<1%) were also found in both the countries. There were seven Asia II 1 haplotypes unique to India and 16 unique to Pakistan. Eight haplotypes of Asia II 5 were present, but only two were from Pakistan. There were seven haplotypes of Asia II 7, six from Pakistan and one from India. Eleven haplotypes were present among the 77 specimens of Asia 1 with the commonest haplotype detected in both the countries. There were nine Asia 1 haplotypes unique to India and one unique to Pakistan. Seven haplotypes were present among the 46 specimens of MEAM 1 with the most common comprising 59% of the total and present only in Pakistan.

**Table 2:** Genetic diversity indices and neutrality tests (Fu’s *Fs* and Tajima’s *D*) in the mtCOI-5′ (barcode) sequences of putative species in *Bemisia tabaci* complex from Pakistan and India.

Species	*n*	S	k	π	Eta(s)	Hd	Fu’s *Fs*	Tajima’s *D*
*Asia 1*	77	13	2.3	0.0036	6	0.645	−1.197	−0.319
*Asia II 1*	551	31	1.93	0.0039	19	0.578	−14.137	−1.48891
*Asia II 5*	22	15	1.82	0.0028	12	0.697	−2.288	−2.00862
*Asia II 7*	14	13	5.53	0.01	2	0.879	0.798	1.41512
*Middle East-Asia Minor 1*	46	8	1.76	0.0034	4	0.602	−0.212	−0.09123

*n*: number of sequences; S: number of polymorphic sites; k: average number of pairwise nucleotide differences; π: nucleotide diversity; Eta(s): total number of singleton mutations; Hd: haplotype (gene) diversity.

Fu’s *Fs:* A negative value of *F_S_* is evidence for an excess number of alleles, as would be expected from a recent population expansion or from genetic hitchhiking. A positive value of *F_S_* is evidence for an deficiency of alleles, as would be expected from a recent population bottleneck. Statistical significance: Not significant, P>0.02.

Tajima’s *D*: A negative Tajima’s *D* signifies an excess of low frequency polymorphisms relative to expectation. A positive Tajima’s *D* signifies low levels of both low and high frequency polymorphisms. Statistical significance: Not significant, P>0.10.

The five species of the *B. tabaci* complex identified through COI-3′ analysis showed marked variation in abundance in Pakistan with Asia II 1 comprising 88%, MEAM 1–7%, Asia II 7–2%, Asia 1–2% and Asia II 5–0.3% of the individuals.

**Figure 7:**
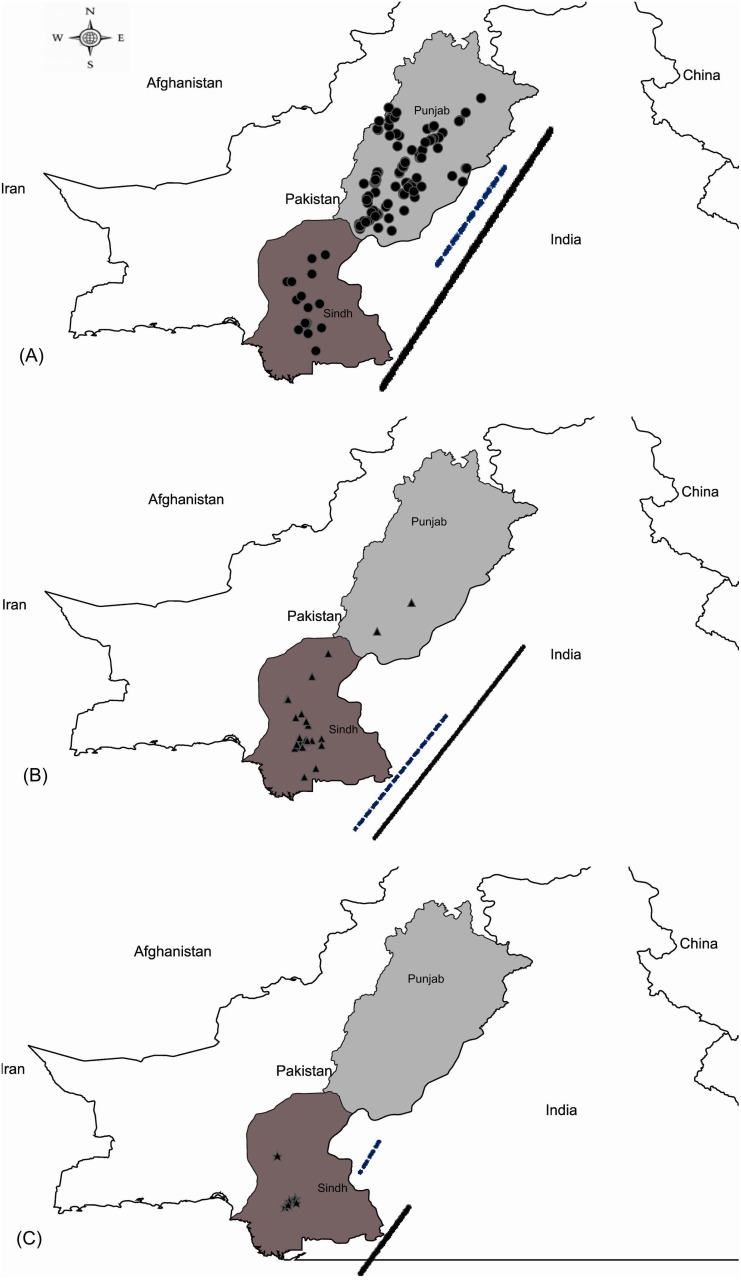
Map of Punjab and Sindh, Pakistan showing the distribution of three species in the *B. tabaci* complex. The range of each species is indicated by a blue broken line (before 2010 [11]) and a black solid line (after 2010 [this study]). (A) *Asia II 1*; (B) *Middle East-Asia Minor 1*; (C) *Asia 1*.

Fig. 7 shows that Asia II 1 was present in all of the cotton-growing areas in Punjab and Sindh. The specimens of Asia II 5 and Asia II 7 derived from central and northern Punjab, while Asia 1 was only present in central and southern Sindh. Finally, MEAM 1 was detected from all three regions (northern, central and southern) of Sindh and from southern Punjab. Chi-square analysis showed a significant heterogeniety for species abundance between the two provinces (χ^2^ = 203; *p* = 0.0) and for species composition (χ^2^ = 856.9; *p* = 0.0) which was clearly skewed towards Asia II 1. The host information on whitefly specimens from India and Pakistan showed that the species of *B. tabaci* complex in Pakistan were recorded on multiple plants (Table 1). Asia II 1, the most frequent whitefly species in Pakistan, was recorded from at least 14 host plants (Table 1).

## Discussion

The varied incidence of cotton leaf curl disease in different areas of Pakistan [73] raises the possibility that disease transmission may be influenced by regional variation in species composition of the *B. tabaci* complex whose member taxa vector the virus responsible for this disease. Although examination of reproductive compatibility among the putative species has also been successful [74], sequence analysis has been frequently used to discriminate members of this complex [1], [21] and COI-3′ has been the standard marker employed for their separation [3], [8], [59]. However, COI-5′ has been adopted as the DNA barcode standard for the entire animal kingdom [34], [35] and its use is gaining adoption for biosecurity [42] and regulation [75]. The superiority of DNA barcoding over traditional methods for the detection and distribution analysis of invasive species is now well established [76], [77]. Despite this fact, the present study represents the first effort to obtain both COI-5′ and COI-3′ sequences for members of the *B. tabaci* complex to develop a correspondence map between haplotypes recognized by these two markers.

Analysis of sequence diversity in COI-5′ revealed that six species of the *B. tabaci* complex were present in Pakistan. Determination of their COI-3′ sequences established that they included Asia II 1, Asia II 5, Asia II 7, Asia 1, MEAM 1, and a new species “Pakistan”. Four of these species (Asia II 1, Asia II 5, Asia 1, MEAM 1) were found on cotton in Pakistan. Asia II 7 was only collected in a Malaise trap in Pakistan, but has been recorded on cotton in India. The levels of sequence divergence at COI-3′ and COI-5′ were generally congruent (data not shown), indicating the interchangeability of the markers. An earlier study of whiteflies from Pakistan [11] revealed three species (Asia II 1, Asia 1, MEAM 1), while our results indicated the presence of three more - one on cotton (Asia II 5) and two from uncertain hosts (Asia II 7 and “Pakistan”, both collected in Malaise traps). The previous two studies on whiteflies in Pakistan examined fewer specimens and fewer geographic localities. Ahmed et al. [17] sequenced 16 specimens from 16 locations, while Ahmed et al., [11] sequenced 141 specimens from 48 locations while this study examined 593 specimens from 255 locations. The most recent study on whitefly diversity [78] reported the presence of three genetic groups in cotton areas of Pakistan, but the sample size was small (80) and the technique used (RAPD) prevents species identification.

The analysis of all currently available COI-5′ data for *B. tabaci* indicated the presence of 15 deeply divergent lineages, including 12 from the Indo-Pakistan region. Multiple genotypes of *B. tabaci* have previously been reported from the Indo-Pakistan subcontinent [11], [17], [79]. Lisha et al. [15] detected two distinct biotypes of *B. tabaci* in India, while Rekha et al. [79] noted three groups - Asia II 5, Asia II 7 and Asia II 8 [3]. More recently, Chowda-Reddy et al. [24] found five species in India (Asia 1, Asia II 5, Asia II 7, Asia II 8, MEAM 1) based on their survey of multiple host plants. In our study, except for Asia II 8, we detected all the species previously identified from India [3], [24] and connected their COI-5′/COI-3′.

The COI-3′ sequence of one whitefly (BIN: ABX2616) from northern Punjab showed 13.7% K2P divergence from any known lineage, indicating that it represents a new species in the *B. tabaci* complex. The NN barcode distances in the *B. tabaci* complex ranged between 1.0%–16.7% with the new putative species, “Pakistan”, showing a NN distance of 14.3%. Further, both the BI and parsimony analysis showed that the “Pakistan” lineage formed a separate branch on the tree and was phylogenetically closer to species in the African group. Previous researchers have used genetic distances and phylogenetic analysis to determine the relationships and taxonomic status of species of the *B. tabaci* complex [3], [5], [7], and the number of species in this complex has generally been assessed by BI [3], [7].

It has been established that begomovirus spread and diversification is linked to the genetic and phenotypic variability of whiteflies [16]. We analysed the genetic diversity in whiteflies at sites across Punjab and Sindh to see if there was any correspondence with the varying incidence of CLCuD in these regions as found in an earlier study in Africa [80]. Two previous studies which examined genetic diversity in *B. tabaci* across Punjab and Sindh from 2007 to 2009 [11], [17] found that MEAM 1 was widespread across Sindh, but absent from Punjab. However, our study detected this species in southern Punjab. Ahmed et al. [11] found Asia 1 at sites from central Punjab to northern Sindh, but our studies indicated that it is now restricted to central and southern Sindh. Ahmed et al. [11], [17] found that Asia II 1 was prevalent througout Punjab, but absent from Sindh. Our study revealed that it remains the commonest species in Punjab, but that it is now also the dominant species on cotton in Sindh, revealing that it has expanded its range to the south. Asia II 5 was only detected in two districts in central and northern Punjab, and Asia II 7 only in northern Punjab, but these are the first records for these species in Pakistan. Other studies have reported the rapid displacement of one whitefly species by another [29], [30], [81], [82]. For example, Guo et al. [19] reported that MEAM 1 was dominant in most provinces of China prior to 2007, but that the Mediterranean (MED) species was now dominant species in at least 11 provinces.

The prevalence of Asia II 1 in Sindh is important because Ahmed et al. [11] observed that a higher incidence of CLCuD was associated with this species. If its greater vector competence compared with other members of the complex is confirmed, then the Sindh detections likely signal an increased threat and there are reports [12], [83] of increased CLCuD in the cotton areas of Sindh. The prevalence and epidemiology of CLCuD in cotton-growing areas of Punjab is well studied [84] and the role of Asia II 1 in the spread of CLCuD has been documented [11], [17]. However, further work is needed to assess temporal shifts in the abundance and distribution of species in the *B. tabaci* complex to validate these impacts. Experimental assessment of vector competence for each member of the complex as well as evaluation of their host preference would also improve understanding of the epidemiology of CLCuD in Pakistan.

Species in the *B. tabaci* complex within Pakistan seem to show the same dynamic distributional shifts detected in other regions [23], [26], [29], a factor which might influence the incidence of begomoviruses. However, knowledge of CLCuD in Pakistan lacks sufficient quantitative data to understand the epidemiology of this disease with vector composition.

## Supporting Information

Table S1 BOLD process IDs, GenBank accessions, collection locations and host plants of *Bemisia tabaci* included in the study.
(XLS)

Table S2 COI-3′ and COI-5′ (barcode) K2P distances among respective taxonomic units of *Bemisia tabaci* complex as determined by Dinsdale et al. [3] and Ratnasingham and Hebert [54].
(XLS)
